# Herbicide resistance in *Leptochloa chinensis* (L.) Nees populations from different regions of Jiangsu Province, China: sensitivity differences and underlying mechanisms

**DOI:** 10.3389/fpls.2025.1535877

**Published:** 2025-02-04

**Authors:** Peng Xu, Ke Wang, Yawen Ju, Yousheng Fu, Axiu Zhu, Kaige Cao, Hongchun Wang

**Affiliations:** ^1^ Huaiyin Institute of Agriculture Science of Xuhuai Area in Jiangsu, Huai’an, China; ^2^ Institute of Plant Protection, Jiangsu Academy of Agricultural Sciences, Nanjing, China; ^3^ Plant Protection and Quarantine Station of Jiangsu Province, Nanjing, China

**Keywords:** *Leptochloa chinensis* (L.) Nees, geographical populations, herbicide, cyhalofop-butyl, sensitivity, target mutation

## Abstract

*Leptochloa chinensis* (L.) Nees, a noxious weed species commonly found in rice fields, has become a significant challenge in Jiangsu Province, China, as it has developed resistance to multiple herbicides due to extensive and continuous herbicide use in recent years. Therefore, this study was conducted to elucidate sensitivity differences and the mechanisms underlying the resistance of *L. chinensis* (L.) Nees populations to commonly used herbicides across different regions of Jiangsu Province, China. A whole-plant bioassay was used to assess the sensitivity of 46 *L. chinensis* populations collected from various areas within Jiangsu to several herbicides frequently applied in paddy fields, including: cyhalofop-butyl, fenoxaprop-P-ethyl, pyraclonil, benzobicyclon, anilofos, and oxaziclomefone. After treatment with cyhalofop-butyl, 38 out of 46 populations showed relative resistance-index values that were over four times that of the controls, indicating significant resistance to cyhalofop-butyl. All 41 cyhalofop-butyl-resistant populations showed cross-resistance to fenoxaprop-P-ethyl but remained susceptible to pyraclonil, benzobicyclon, anilofos, and oxaziclomefone. The proportion of populations resistant to acetyl-CoA carboxylase (ACCase)-inhibiting herbicides increased progressively from the south to the north of Jiangsu. Cross-resistance was evident between cyhalofop-butyl and fenoxaprop-P-ethyl; however, all resistant populations were susceptible to pyraclonil, benzobicyclon, anilofos, and oxaziclomefone. Furthermore, mutations in the *ACCase* gene were identified as a crucial mechanism for cyhalofop-butyl resistance. Specifically, we found *ACCase* mutations I1781L, W1999C, W2027C/L/S, I2041N, and D2078G in cyhalofop-butyl-resistant *L. chinensis* populations, among which, W1999C and W2027C accounted for a relatively high proportion, while I1781L, W2027L/S, I2041N, and D2078G were found in one population each. *ACCase* gene mutations are seemingly a key mechanism for the development of resistance to cyhalofop-butyl, thus, our study provides useful information for developing effective weed-management strategies for controlling this noxious weed species, while ensuring sustainable agricultural practices.

## 1 Introduction

*Leptochloa chinensis* (L.) Nees is a tetraploid (2n=4x=40) belonging to the Chloridoideae subfamily of the Poaceae(grass) family, with high drought and waterlogging resistance, moisture preference, and strong reproductive and tillering abilities. Furthermore, this grass species is globally recognized as a noxious agricultural weed (Chauhan and Johnson, 2008; Wen et al., 2017; Zhu et al., 2018). The spread and harmful effect of *L. chinensis* in the middle and lower reaches of the Yangtze River Basin in China have shown a clear increasing trend over time, which has been exacerbated by the widespread adoption of direct-seeded rice cultivation practices and prolonged, exclusive use of herbicides, posing a severe threat to rice production security (Wu et al., 2015; Yuan et al., 2022).

Cyhalofop-butyl is an acetyl-CoA carboxylase (ACCase, EC.6.4.12) inhibitor herbicide traditionally used in paddy fields for the management of Poaceae weeds, including *L. chinensis* and *Echinochloa crus-galli* (Ruiz-Santaella et al., 2006). However, the efficacy of cyhalofop-butyl against *L. chinensis* has been declining owing to its long-term, exclusive use. This has led to the development of resistance among weeds, as indicated by an increasing number of reports from farmers stating that recommended doses are ineffective in controlling the weed. Exclusive use of ACCase inhibitor herbicides over extended periods can induce resistance in weeds. To date, 49 species of weeds have reportedly developed resistance to this class of herbicides globally (Heap, 2024; Heap and Knight, 1986; Maneechote et al., 2005). Thus, a population of *L. chinensis* resistant to ACCase inhibitors, including cyhalofop-butyl, was identified in Bangkok, Thailand in 2002 (Heap, 2024). Similarly, *L. chinensis* populations resistant to cyhalofop-butyl were detected in the paddy fields of Hubei Province, China in 2011; *L. chinensis* populations showing cross-resistance to cyhalofop-butyl and fenoxaprop-P-ethyl were reported from South Korea in 2012 (Sahid et al., 2011; Wei et al., 2015), and *L. chinensis* populations resistant to cyhalofop-butyl were identified in Malaysia in 2014 (Rahman et al., 2014). Currently, *L. chinensis* populations resistant to cyhalofop-butyl have been identified in Jiangsu (Deng et al., 2019), Shanghai (Yuan et al., 2022), Anhui (Jiang et al., 2022), and Hunan (Peng et al., 2020). Several mutations, such as Ile-1781-Leu, Leu-1818-Phe, Trp-1999-Cys, Trp-2027-Cys, Trp-2027-Gly, Trp-2027-Leu, Trp-2027-Ser, Ile-2041-Asn, Cys-2088-Arg, and Gly-2096-Ala, in ACCase have been identified as the primary cause of *L. chinensis* resistance (Kurniadie et al., 2022; Zhang et al., 2022; Zhao et al., 2022; Liao et al., 2024; Deng et al., 2023; Jiang et al., 2024).

In turn, Deng et al. (2019) detected cyhalofop-butyl-resistant populations of *L. chinensis* in the paddy fields of Jiangsu Province in 2019. However, variation in sensitivity to cyhalofop-butyl and other commonly used herbicides among different geographic populations of *L. chinensis* in Jiangsu, along with their distribution and the underlying mechanisms responsible for resistance, remain unclear. Thus, in this study, we systematically collected seed samples of *L. chinensis* from paddy fields across the southern, central, and northern regions of Jiangsu Province to test their sensitivity to the commonly used herbicides, the aryloxyphenoxypropionate(APP) family of ACCase inhibitor cyhalofop-butyl(HRAC 1, inhibition of Acetyl CoA Carboxulase) and fenoxaprop-P-ethyl(HRAC 1, inhibition of Acetyl CoA Carboxulase), the protoporphyrinogen oxidase(PPO) inhibitor pyraclonil(HRAC 14, inhibition of Protoporphyrinogen Oxidase), the 4-hydroxyphenylpyr­uvate dioxygenase inhibitor benzobicyclon(HRAC 27, inhibition of Hydroxyphenyl Pyruvate Dioxygenose), the organophosphorus herbicide anilofos(HRAC 15, inhibition of Very Long-Chain Fatty Acid Synthesis), and the organoheterocyclic herbicide oxaziclomefone(HRAC 30, inhibition of Fatty Acid Thioesterase), with the aim to analyze the distribution of resistant populations and the key molecular mechanisms responsible for resistance. Our study aimed to provide technical guidance for the effective management and control of *L. chinensis* in paddy fields.

## 2 Materials and methods

### 2.1 Seeds

Seeds were randomly collected from various locations across southern and northern Jiangsu Province during seed maturation period, from September to November 2020. The LENJ-001 population is a known susceptible population. Specific collection sites are listed in **Table 1**.

**Table 1:** *Leptochloa chinensis* (L.) Nees seed collection sites.

Order	Population code	Site of collection	Geographical location
1	LESZ-001	Tongli Town, Wujiang District, Suzhou City	31°6′N, 120°43′E
2	LESZ-003	Guli Town, Changshu County, Suzhou City	31°40′N, 120°49′E
3	LESZ-005	Daxin Town, Zhangjiagang City, Suzhou City	31°57′N, 120°31′E
4	LEJY-001	Songjiawei Village, Jiangyin City, Wuxi City	31°52′N, 120°7′E
5	LEHS-003	Yuqi Street, Huishan District, Wuxi City	31°42′N, 120°10′E
6	LEYX-003	Dingshu Town, Yixing City, Wuxi City	31°16′N, 119°50′E
7	LELY-003	Nandu Town, Liyang City, Changzhou City	31°26′N, 119°19′E
8	LEJT-001	Zhiqian Town, Jintan District, Changzhou City	31°39′N, 119°28′E
9	LELS-001	Baima Town, Lishui District, Nanjing City	31°34′N, 119°10′E
10	LEJR-001	Shishi Town, Jurong City, Zhenjiang City	31°56′N, 119°9′E
11	LEJR-002	Guozhuang Town, Jurong City, Zhenjiang City	31°48′N, 119°1′E
12	LELH-003	Chengqiao Town, Liuhe District, Nanjing City	32°22′N, 118°43′E
13	LEYZ-004	Qianjin Village, Yizheng City, Yangzhou City	32°18′N, 119°4′E
14	LEYL-001	Yiling Town, Jiangdu District, Yangzhou City	32°28′N, 119°40′E
15	LEJD-001	Daqiao Town, Jiangdu District, Yangzhou City	32°21′N, 119°42′E
16	LEJD-003	Dinggou Town, Jiangdu District, Yangzhou City	32°33′N, 119°39′E
17	LEGY-001	Ganduo Town, Gaoyou City, Yangzhou City	32°49′N, 119°46′E
18	LEGY-002	Ganduo Town, Gaoyou City, Yangzhou City	32°50′N, 119°44′E
19	LERG-001	Changjiang Town, Rugao City, Nantong City	32°6′N, 120°36′E
20	LERG-002	Wuyao Town, Rugao City, Nantong City	32°12′N, 120°32′E
21	LERG-003	Wuyao Town, Rugao City, Nantong City	32°12′N, 120°32′E
22	LEJJ-001	Dongxing Town, Jingjiang City, Taizhou City	31°58′N, 120°9′E
23	LETX-002	Huangqiao Town, Taixing City, Taizhou City	32°14′N, 120°14′E
24	LEXH-002	Xingdong Town, Xinghua City, Taizhou City	32°58′N, 119°54′E
25	LEXH-003	Diaoyu Town, Xinghua City, Taizhou City	33°4′N, 119°58′E
26	LEXH-006	Diduo Town, Xinghua City, Taizhou City	32°50′N, 120°5′E
27	LEHQ-001	Hongqi Farm, Jiangyan District, Taizhou City	32°33′N, 120°1′E
28	LEDF-007	Fangqiang Farm, Dafeng District, Yancheng City	33°29′N, 120°28′E
29	LESY-005	Haitong Town, Sheyang County, Yancheng City	33°48′N, 120°20′E
30	LEFN-001	Chenliang Town, Funing County, Yancheng City	33°41′N, 119°56′E
31	LEHX-001	Huanghai Farm, Xiangshui County, Yancheng City	34°17′N, 120°1′E
32	LEHX-002	Huanghai Farm, Xiangshui County, Yancheng City	34°17′N, 120°1′E
33	LEBH-001	Binhuai Farm, Binhai County, Yancheng City	34°15′N, 120°6′E
34	LEHH-001	Huaihai Farm, Sheyang County, Yancheng City	34°9′N, 120°14′E
35	LESQ-001	Wangji Town, Siyang County, Suqian City	33°52′N, 118°44′E
36	LESQ-002	Chuancheng Town, Siyang County, Suqian City	33°36′N, 118°37′E
37	LESQ-003	Lailong Town, Suyu District, Suqian City	34°2′N, 118°29′E
38	LESN-001	Daodao, Weiji Town, Suining County, Xuzhou City	34°1′N, 117°57′E
39	LESN-002	Weiji Town, Suining County, Xuzhou City	34°1′N, 117°57′E
40	LELSH-001	Gaozhuang, Lianshui County, Huai’an City	33°58′N, 119°5′E
41	LEBYH-001	Huai’an Baoying Lake Farm	33°36′N, 119°1′E
42	LEGY-001	Songzhuang Town, Ganyu District, Lianyungang City	34°46′N, 119°8′E
43	LEGYD-001	Dongxin Farm, Guanyun County, Lianyungang City	34°35′N, 119°22′E
44	LEYT-002	Yuntai Farm, Haizhou District, Lianyungang City	34°39′N, 119°23′E
45	LEDX-003	Dongxin Farm, Lianyun District, Lianyungang City	34°32′N, 119°22′E
46	LENJ-001	Xuanwu District, Nanjing City	30°2’N, 118°52’E

### 2.2 Herbicide treatments

Test chemicals and the manufacturers are shown in **Table 2**. The technical materials of cyhalofop -butyl, fenoxaprop-P-ethyl, pyraclonil, benzobicyclon, anilofos, and oxaziclomefone were individually dissolved in 1mL acetone and then diluted to a series of solutions with water containing 0.1% (v/v) Tween-80 aqueous solution.

**Table 2:** Herbicides and manufacturers.

Herbicide	Test doses (g a.i. ha^-1^)	Manufacturer
98% Cyhalofop-butyl TC	105	Jiangsu Agrochem Laboratory Co., Ltd., Jiangsu, China
96% Fenoxaprop-P-ethyl TC	37.5	Jiangsu Agrochem Laboratory Co., Ltd., Jiangsu, China
97% Pyraclonil TC	210	Hubei Xianghe Precision Chemistry Co., Ltd., Hubei, China
98% Benzobicyclon TC	225	SDS Biotech K.K., Tokyo, Japan
95% Anilofos TC	315	Shandong Binnong Technology Co., Ltd., Shandong, China
97% Oxaziclomefone TC	45	Jiangsu Agrochem Laboratory Co., Ltd., Jiangsu, China

TC, Technical Material.

### 2.3 Test methods

#### 2.3.1 Cultivation of the experimental materials

Whole-plant bioassay was used in this experiment. White plastic pots (7 cm in length and width and 9 cm in height), with holes in the bottom, were filled to approximately three-quarters of their capacity with air-dried fine previously sieved soil and placed inside a plastic turnover box. Water was added to the bottom to ensure the soil was saturated with moisture. The soil used for the tests was a loam (pH 6.7) with 1.6% organic matter content. The seeds were sown separately, covered with a thin layer of soil of approximately 0.2–0.3-cm thick and cultivated in a solar greenhouse (temperature: daytime, 25 ± 5°C, nighttime, 25 ± 5°C; light regime: 12-:12-h light/dark). Once the weeds had emerged uniformly, seedlings were thinned to 12 per pot.

#### 2.3.2 Determination of the sensitivity of *L. chinensis* populations to cyhalofop-butyl

When plants reached the 2–3-leaf stage, stem and leaf spray treatments were applied. Spraying was performed with a 3WPSH-500D bioassay spray tower developed by the Nanjing Institute of Agricultural Mechanization of the Ministry of Agriculture, with a disk diameter of 50 cm, a spindle rotation speed of 6 rpm, nozzle aperture of 0.3 mm, spray pressure of 0.3 MPa, droplet diameter of 100 μm, and a nozzle flow rate of 90 mL/min. The volume of water used was 450 L ha^-1^. There were six experimental doses: 1/4, 1/2, 1, 2, 4, and 8 times the upper limit of the recommended field application rate of cyhalofop-butyl, which is 105 g a.i.ha^-1^, resulting in doses of 26.25, 52.5, 105, 210, 420, and 840 g a.i. ha^-1^, respectively. The herbicide doses were 6.56, 13.12, 26.24, 52.48, 104.96, and 209.92 g a.i. ha^-1^ for the LENJ-001 population. For each *L. chinensis* population, an untreated blank control was set up, and four replications per treatment were included. At 20 d after treatment, the fresh weight of the aerial parts of the *L. chinensis* plants was measured. Each treatment had four replicates, and the experiment was repeated twice. Excel Software was used to analyze the inhibition of biomass accumulation in the areal plant body parts. The statistical analysis software SigmaPlot v.14.0 (Systat Software, San Jose, CA, USA) was used to perform a four parameter double logistic nonlinear regression analysis on the data using the formula y = C+(D-C)/[1+(x/GR_50_)^b^] and calculate the herbicide dosage required to inhibit 50% plant growth (GR_50_). In the formula, x is the specific herbicide dosage, y is the relative fresh weight of weeds under x treatment, C is the lower response limit; D is the upper response limit, and b is the slope of the curve at GR_50_. Relative resistance index (RI) was calculated as the GR_50_ of the resistant population divided by the GR_50_ of the susceptible population. RI ≤ 2 indicates a susceptible population (S), 2 < RI ≤ 5 indicates low resistance (LR), 5 < RI ≤ 10 indicates moderate resistance (MR), and RI > 10 indicates high resistance (HR).

#### 2.3.3 Analysis of *ACCase* mutations in *L. chinensis* populations

Based on the results of the cyhalofop-butyl-resistance test above, all populations were selected, with at least 10 plants per population. Fresh leaf tissue was sampled for genomic DNA extraction. Primers were designed according to the method described by Jiang et al. (2022) (F: 5′-GTGGTGAGGTTATTTGGATTATTGAC-3′; R: 5′-CACCTTGGAGTTTTGCTTTCAG-3′), covering seven amino acid variation sites known to contribute to *ACCase* inhibitors resistance (I1781, W1999, W2027, I2041, D2078, C2088, and G2096). The target fragment with 1128 bp was amplified using TaKaRa LA Taq polymerase for PCR under the following conditions: initial denaturation at 98°C for 3 min; 35 cycles at 98°C for 30 s, 56°C for 30 s, and 72°C for 30 s; followed by a final extension at 72°C for 10 min. Subsequently, the polymerase chain reaction (PCR) products were separated using electrophoresis, recovered and purified using agar gel with TIAN gelMidi Purification Kit (Tiangen, Beijing, China). The purified products were cloned into the pMD20-T vector (TaKaRa Biotechnology). The recombinant plasmids containing the gene were extracted and sequenced. In order to distinguish the genes encoding plasticity ACCase in *L. chinensis*, at least 20 clones were randomly selected from each biological replicate for sequencing. The resulting sequences were aligned and compared using DNAMAN v 6.0 software (Lynnon, Quebec, Canada).

#### 2.3.4 Determination of the level of sensitivity of *L. chinensis* to other herbicides

When plants reached the 2–3-leaf stage, the stems and leaves were sprayed using a 3WPSH-500D bioassay spray tower described above. Information on the herbicides used in the whole-plant doseresponse experiments are showed in **Table 2**. An untreated blank control was prepared for each *L. chinensis* population; each treatment included four replicates and the experiment was conducted in duplicate. At 20 d after treatment, fresh weight of the aerial parts of *L. chinensis* was measured, and the rate of inhibition of biomass accumulation was analyzed using Microsoft Excel.

## 3 Results

### 3.1 Differences in the sensitivity of *L. chinensis* populations to cyhalofop-butyl

**Figure 1:**
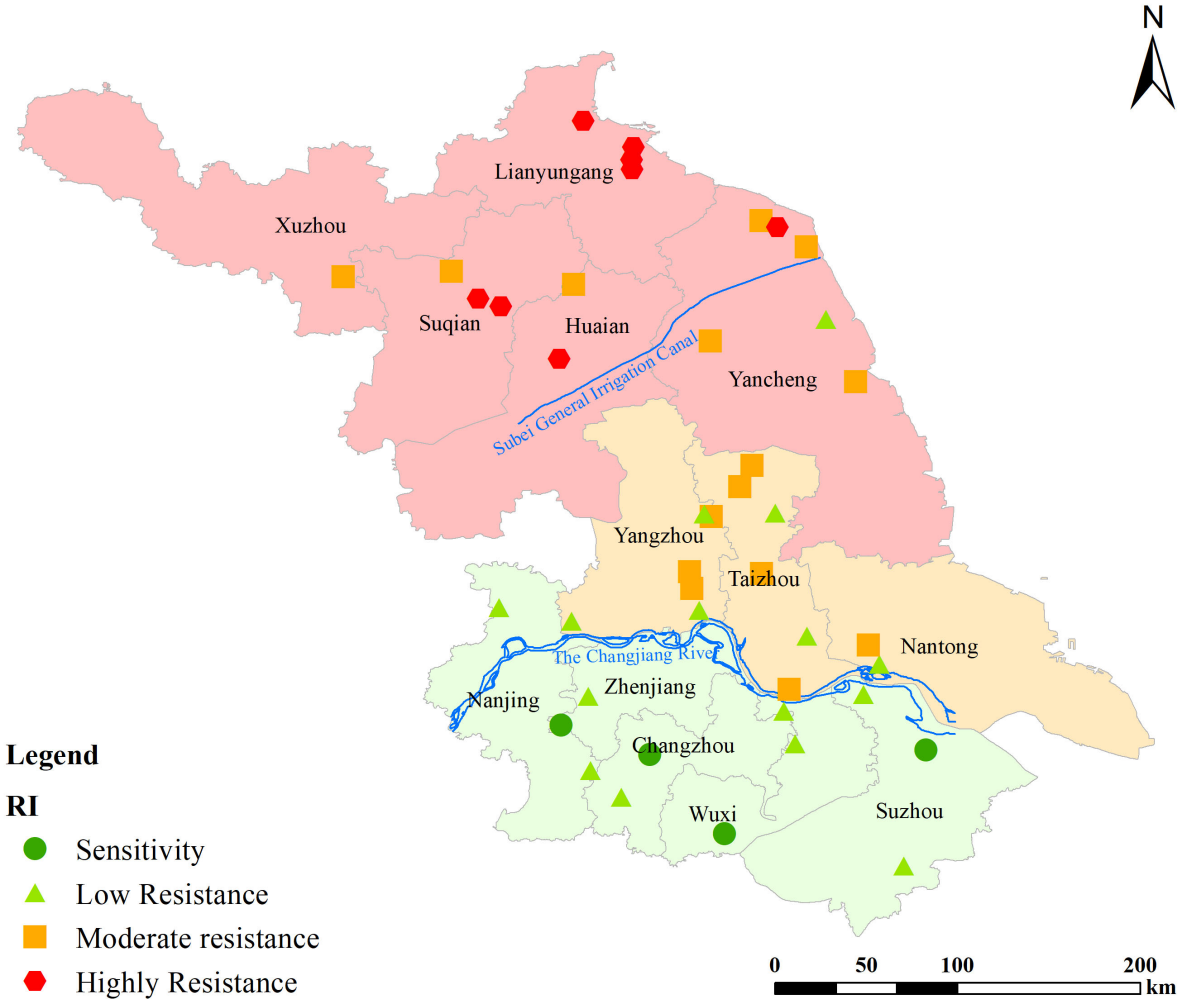
Distribution of different geographical populations of *Leptochloa chinensis* (L.) Nees in Jiangsu Province, China.

A whole-plant bioassay was used to determine the sensitivity of 46 *L. chinensis* populations collected from various areas of Jiangsu Province to cyhalofop-butyl, a commonly used aryloxyphenoxypropionate herbicide in paddy fields. The results (**Table 3**) indicated that 41 out of the 46 *L. chinensis* populations exhibited 2.07- to 14.58 times resistance to cyhalofop-butyl. Although the GR50 values of LESE-001, LESZ-005, LEJY-001, LELY-001 populations were lower than 50% of the field dose, they were still classified as LR. Several researchers think that if for 50% growth inhibition of the population less than ½ of the recommended herbicide dose is sufficient, then it is not a resistant population. Based this rule, the LESE-001, LESZ-005, LEJY-001, LELY-001 populations should be classified as S. The area south of the Yangtze River in Jiangsu Province was defined as the southern Jiangsu region, the area from the Yangtze River to the northern Jiangsu main irrigation canal was defined as the Central Jiangsu region, and the area north of the northern Jiangsu main irrigation canal was defined as the northern Jiangsu region (**Figure 1**). Analysis of the distribution of herbicide resistance across different geographic populations in Jiangsu Province revealed that 7 out of 11 populations in the southern Jiangsu region had 2.07- to 4.96 times resistance, with 63.64% of the populations showing resistance. Meanwhile, in the Central Jiangsu region, all 19 populations showed 4.96- to 11.09 times resistance to cyhalofop-butyl, with 100% of the populations being resistant. In turn, in the Northern Jiangsu region, all 15 populations showed 5.57- to 14.58 times resistance, i.e., again, 100% of the populations were resistant.

**Table 3:** Sensitivity of different geographical populations of *Leptochloa chinensis* (L.) Nees, to cyhalofop-butyl.

Entry	Population	Logistic equation	Correlation coefficient	GR_50_ g a.i. ha^-1^	Susceptibility	Mutation	Frequency of plants with mutation(s) %	RI*
1	LESZ-001	y=-9.1964 + 109.4264/ (1+(x/37.0971) ^-2.2368^)	1.0000	37.0971	LR	WT	–	2.53
2	LESZ-003	y=-53.6186 + 154.2167/(1+(x/23.7359) ^-1.7999^)	0.9999	23.7359	S	–	–	1.62
3	LESZ-005	y=-17.2467 + 117.5376/(1+(x/30.3213)^-1.9665^)	0.9999	30.3213	LR	WT	–	2.07
4	LEJY-001	y=6.8889 + 93.1705/ (1+(x/39.2875) ^-2.6445^)	1.0000	39.2875	LR	WT	–	2.68
5	LEHS-003	y=25.2840 + 74.8558/ (1+(x/63.3494)^-4.3050^)	1.0000	63.3494	LR	WT	–	4.32
6	LEYX-003	y=-28.2482 + 128.5793/(1+(x/26.9161)^-1.7531^)	0.9993	26.9161	S	–	–	1.83
7	LELY-003	y=-10.4796 + 110.8085/(1+(x/31.4017)^-2.0106^)	1.0000	31.4017	LR	WT	–	2.14
8	LEJT-001	y=-94.2302 + 194.3745/(1+(x/18.5684)^-1.7307^)	0.9990	18.5648	S	–	–	1.27
9	LELS-001	y=16.7391 + 78.8823/ (1+(x/79.5397)^-3.4533^)	0.9902	79.5397	MR	W2027C	90	5.42
10	LEJR-001	y=11.9962 + 82.8761/ (1+(x/72.7335)^-2.5779^)	0.9833	72.7335	LR	WT	–	4.96
11	LEJR-002	y=-129.5258 + 229.5656/ (1+(x/16.1023)^-1.6053^)	0.9965	16.1023	S	–	–	1.10
12	LELH-003	y=14.6115 + 80.9054/ (1+(x/73.0230)^-3.4343^)	0.9921	73.0230	LR	WT	–	4.98
13	LEYZ-004	y=18.1072 + 76.2527/ (1+(x/87.7839)^-5.4614^)	0.9861	87.7839	MR	W2027C	100	5.98
14	LEYL-001	y=8.4036 + 79.0351/ (1+(x/82.1600)^-3.5896^)	0.9651	82.1600	MR	W2027C	100	5.60
15	LEJD-001	y=9.4320 + 88.7853/ (1+(x/72.8151)^-2.2299)	0.9966	72.8151	LR	WT	–	4.96
16	LEJD-003	y=-5.4165 + 101.7864/ (1+(x/104.7670)^-1.2963^)	0.9928	104.7670	MR	W2027C	100	7.14
17	LEGY-001	y=7.7252 + 84.6088/ (1+(x/75.8842)^-2.6572^)	0.987	75.8842	MR	W2027L	80	5.17
18	LEGY-002	y=8.3153 + 87.4225/ (1+(x/77.1450)^-2.5675^)	0.9898	77.1450	MR	I2041N	70	5.26
19	LERG-001	y=9.0839 + 85.7091/ (1+(x/85.0290)^-2.4508^)	0.9788	85.0290	MR	W1999C	70	5.79
20	LERG-002	y=6.5286 + 92.8018/ (1+(x/82.4145)^-1.8677^)	0.9875	82.4145	MR	WT	–	5.62
21	LERG-003	y=-0.7142 + 93.4364/ (1+(x/102.3075)^-1.6654^)	0.9918	102.3075	MR	W2027C	100	6.97
22	LEJJ-001	y=-12.4920 + 104.4448/(1+(x/82.3830)^-1.3574^)	0.9800	82.3830	MR	I1781L	90	5.61
23	LETX-002	y=11.9585 + 81.4586/ (1+(x/76.1895)^-3.6082^)	0.9846	76.1895	MR	W2027C	90	5.19
24	LEXH-002	y=4.7908 + 84.3971/ (1+(x/82.0110)^-2.8081^)	0.9862	82.0110	MR	W1999C	90	5.59
25	LEXH-003	y=6.6055 + 84.8186/ (1+(x/102.8400)^-2.3092^)	0.9964	102.8400	MR	W2027C	100	7.01
26	LEXH-006	y=6.1199 + 95.4984/ (1+(x/74.3895)^-1.6790^)	0.9986	74.3895	MR	W1999C	90	5.07
27	LEHQ-001	y=7.6368 + 82.5764/ (1+(x/83.4090)^-2.7359^)	0.9876	83.4090	MR	W2027C	100	5.68
28	LEDF-007	y=4.5508 + 93.8466/ (1+(x/162.8010)^-1.7952^)	0.9995	162.8010	HR	W2027S	100	11.09
29	LESY-005	y=7.2280 + 88.2909/ (1+(x/81.0360)^-3.1872^)	0.9928	81.0360	MR	W2027C	90	5.52
30	LEFN-001	y=0.9214 + 93.8226/ (1+(x/81.9435)^-1.8817^)	0.9978	81.9435	MR	W2027C	100	5.58
31	LEHX-001	y=3.6921 + 84.0791/ (1+(x/167.4600)^-1.9868^)	0.9995	167.4600	HR	W2027C	100	11.41
32	LEHX-002	y=-1.0350 + 105.9160/ (1+(x/156.9210)^-1.2119^)	0.9995	156.9210	HR	W1999C	100	10.69
33	LEBH-001	y=7.8959 + 84.2202/ (1+(x/207.4485)^-2.0099^)	0.9979	207.4485	HR	W2027C	100	14.14
34	LEHH-001	y=1.9036 + 95.0790/ (1+(x/150.8805)^-1.6903^)	0.9976	150.8805	HR	W2027C	90	10.28
35	LESQ-001	y=7.2445 + 87.2385/ (1+(x/149.8895)^-1.7711^)	0.9976	149.8995	HR	W2027C	100	10.21
36	LESQ-002	y=-2.6873 + 121.1010/ (1+(x/213.9915)^-0.9235^)	0.907	213.9915	HR	W2027C	100	14.58
37	LESQ-003	y=6.3374 + 88.2822/ (1+(x/121.3575)^-1.6015^)	0.9878	121.3575	MR	W1999C	100	8.27
38	LESN-001	y=4.5054 + 93.4969/ (1+(x/106.6470)^-1.5236^)	0.998	106.6470	MR	WT	–	7.27
39	LESN-002	y=-26.3543 + 138.2501/(1+(x/97.4535)^-0.8147^)	0.9994	97.4535	MR	W1999C	100	6.64
40	LELSH-001	y=-16.0025 + 116.9730/(1+(x/81.7770)^-1.1393^)	0.9951	81.7770	MR	WT	–	5.57
41	LEBYH-001	y=6.5731 + 85.5943/ (1+(x/202.1970)^-1.7603^)	0.9985	202.1970	HR	W1999C	100	13.78
42	LEGY-001	y=1.4817 + 94.8060/ (1+(x/147.0945)^-1.3893^)	0.9973	147.0945	HR	W2027C	100	10.02
43	LEGYD-001	y=4.1636 + 95.5794/ (1+(x/203.1885)^-1.5127^)	0.9963	203.1885	HR	W1999C	100	13.85
44	LEYT-002	y=-6.0716 + 101.6062/ (1+(x/121.9425)^-1.3588^)	0.9993	121.9425	HR	D2078G	100	8.31
45	LEDX-003	y=-7.9602 + 104.9971/ (1+(x/126.5910)^-1.1405^)	0.9998	126.5910	HR	W2027	100	8.63
46	LENJ-001	y=-167.1343 + 267.6653/(1+(x/14.6750)^-1.7750^)	0.9999	14.6750	S			

*Resistance index value.

### 3.2 Analysis of *ACCase* mutations in resistant population of *L. chinesis*

An amplified sequence fragment of 1,128 bp including seven known mutation sites showed >96% homology with the corresponding genes of *Alopecurus myosuroides*, a species closely related to *L. chinensis*. A comparison of the translated amino acid sequences with those of *A. myosuroides* revealed mutations I1781L, W1999C, W2027C/L/S, I2041N, and D2078G in *ACCase* of cyhalofop-butyl-resistant *L. chinensis* populations in the paddy fields of Jiangsu Province (**Table 3**); however, no mutations were detected at the C2088 or G2096 sites. Mutations W1999C and W2027C were relatively prevalent, whereas mutations I1781L, W2027L/S, I2041N, and D2078G were each found in a different population.

No resistance mutations were observed in LR populations with an RI of 4.98 or below. Similarly, no resistance mutations were detected in the LELSH-001 population with an RI of 5.57, or the LESN-001 population, with an RI of 7.27. The LELSH-001 and LESN-001 populations ranked as MR to cyhalofop-butyl without any mutations in the *ACCase* amino acid sequence, suggesting that resistance may be attributed to non-target site mechanisms or enhanced *ACCase* expression. The exact causes of this resistance warrant further investigation (**Table 3**).

### 3.3 Cross resistance and multiple resistance in *L. chinensis* populations

After treatment with fenoxaprop-P-ethyl, another member of the aryloxyphenoxypropionate family of ACCase inhibitor herbicides, at 37.5 g a.i. ha^-1^, the inhibition rates of biomass accumulation in the aerial parts of the LEJY-001, LELY-003, and LEJR-002 populations were all >80%, whereas those of the remaining 43 populations were all <80% (**Table 4**). Among the 30 populations with a biomass inhibition rate of <50% following treatment with fenoxaprop-P-ethyl at 37.5 g a.i. ha^-1^, 15 were from the northern Jiangsu region and 15 from the Central Jiangsu region, accounting for 100% of the total number of populations in northern and 75% in Central Jiangsu, respectively.

**Table 4:** Biomass reduction of different geographical populations of *Leptochloa chinensis* (L.) Nees by herbicide treatment.

Population	Inhibition (%) of biomass accumulation					Population	Inhibition (%) of biomass accumulation				
	Fenoxaprop-P-ethyl	Pyraclonil	Benzobicyclon	Anilofos	Oxaziclomefone		Fenoxaprop-P-ethyl	Pyraclonil	Benzobicyclon	Anilofos	Oxaziclomefone
LESZ-001	71.25	100	100	100	100	LEXH-002	25.45	100	100	100	100
LESZ-003	68.65	100	100	100	100	LEXH-003	36.45	100	100	100	98.75
LESZ-005	75.62	100	100	100	100	LEXH-006	48.25	100	100	100	100
LEJY-001	80.36	100	100	100	100	LEHQ-001	22.35	100	100	100	97.75
LEHS-003	76.95	100	100	100	100	LEDF-007	19.85	100	100	100	100
LEYX-003	66.35	100	100	100	100	LESY-005	35.68	100	100	100	99.85
LELY-003	80.25	100	100	100	100	LEFN-001	42.35	100	100	100	100
LEJT-001	100	100	100	100	100	LEHX-001	12.35	100	100	100	100
LELS-001	60.55	100	100	100	100	LEHX-002	15.47	100	100	100	92.35
LEJR-001	77.50	100	100	100	100	LEBH-001	24.78	100	100	100	100
LEJR-002	100	100	100	100	100	LEHH-001	25.65	100	100	100	90.05
LELH-003	54.45	100	100	100	95.45	LESQ-001	28.35	100	100	100	100
LEYZ-004	45.35	100	100	100	97.20	LESQ-002	15.65	100	100	100	100
LEYL-001	50.35	100	100	100	92.35	LESQ-003	12.85	100	100	100	94.35
LEJD-001	60.21	100	100	100	98.85	LESN-001	34.62	100	100	100	100
LEJD-003	45.85	100	100	100	100	LESN-002	29.63	100	100	100	100
LEGY-001	52.36	100	100	100	95.35	LELSH-001	21.35	100	100	100	100
LEGY-002	44.38	100	100	100	100	LEBYH-001	18.75	100	100	100	97.55
LERG-001	35.86	100	100	100	95.8	LEGY-001	34.15	100	100	100	100
LERG-002	41.25	100	100	100	100	LEGYD-001	40.35	100	100	100	100
LERG-003	33.68	100	100	100	96.85	LEYT-002	25.68	100	100	100	95.8
LEJJ-001	36.85	100	100	100	90.35	LEDX-003	19.68	100	100	100	100
LETX-002	42.85	100	100	100	100	LENJ-001	100	100	100	100	100

Treatment with protoporphyrinogen oxidase-inhibitor herbicide pyraclonil at 210 g a.i. ha^-1^, novel bicyclooctane herbicide benzobicyclon at 225 g a.i. ha^-1^, and organophosphorus herbicide anilofos, at 315 g a.i. ha^-1^ resulted in 100% inhibition of biomass accumulation across all 46 *L. chinensis* populations in Jiangsu Province.

Treatment of *L. chinensis* with 45 g a.i. ha^-1^ of the organoheterocyclic herbicide oxaziclomefone at the two-leaf stage led to <97% inhibition of biomass accumulation in 10 out of the 46 *L. chinensis* populations collected from Jiangsu Province, including six populations located in Central and four in northern Jiangsu, whereas the other 36 populations showed 100% inhibition of biomass accumulation.

## 4 Discussion

Cyhalofop-butyl and fenoxaprop-P-ethyl are ACCase inhibitor herbicides (Wei et al., 2015) that have been used continuously for many years in Jiangsu Province as common herbicidal options for controlling Poaceae weeds in paddy fields. In this study, we found that 41 out of the 46 geographical populations of *L. chinensis* present in Jiangsu Province have developed significant resistance to cyhalofop-butyl, and 43 showed significant cross-resistance to fenoxaprop-P-ethyl, with resistance rates reaching 91%–93%. This explains the failure of conventional recommended dosages of cyhalofop-butyl and fenoxaprop-P-ethyl in effectively controlling *L. chinensis* infestations in many areas of Jiangsu Province. The results of resistance distribution of different geographical populations in Jiangsu Province showed that 63.64% of the resistance populations were in southern Jiangsu Province. In the middle of Jiangsu Province, the resistant population accounted for 100%. In northern Jiangsu, the resistant population of *L. chinensis* accounted for 100%. Furthermore, we observed a gradual increase trend in the proportion of *L. chinensis* populations resistant to *ACCase* inhibitor herbicides from the southern to the northern region of Jiangsu, possibly owing to the higher frequency of use of these herbicides in the latter (Zhu et al., 2024).

Research evidence indicates that target-site resistance mechanisms, such as mutations or increased expression of *ACCase*, along with non-target-site resistance mediated by enhanced metabolism, are the two primary mechanisms underlying weed resistance to ACCase inhibitor herbicides (Han et al., 2016; Wang et al., 2013). Often, target-site mutations within *ACCase* are the principal cause of herbicide resistance (Beckie and Tardif, 2012). Thus, to date, 14 types of amino acid mutations at seven amino acid sites within the ACCase carboxyltransferase (CT) domain have been identified in weeds that are resistant to ACCase inhibitor herbicides, namely, I1781L, I1781V, I1781T, W1999C/S/L, W2027C, I2041N, I2041N, D2078G, C2088R, G2096A, and G2096S (Beckie and Tardif, 2012; Délye et al., 2005; Guo et al., 2017; Powles and Yu, 2010). In this study, we identified only previously reported resistance mutations within the ACCase CT domain of resistant *L. chinensis* populations and did not detect any new type of mutation. Previous research has shown that mutations at multiple sites can coexist within the same resistant weed species. For instance, Zhang and Powles (2006) reported six different amino acid mutations in the ACCase CT domain of resistant Italian ryegrass, while Yu et al. (2013) found three distinct *ACCase* mutations on a single plant of wild oats. Furthermore, Phongphitak et al. (2014) conducted a cross-breeding experiment with fenoxaprop-P-ethyl-resistant *L. chinensis* in Kedah, Malaysia, and found that resistance in this case might be controlled by a single gene. Similarly, Yu et al. (2017) discovered a mutation from tryptophan to cysteine at site 2027 in *L. chinensis* populations highly resistant to cyhalofop-butyl, marking the first case of detection of a target-gene mutation in this weed species, with the mutation rate exceeding 50%. Additionally, Wu et al. (2019) identified a replacement of tryptophan with serine at site 1999 in the *ACCase* CT domain of cyhalofop-butyl-resistant *L. chinensis* populations in paddy fields in East China, possibly one of the important factors leading to the observed resistance. So far, Ile-1781-Leu, Leu-1818-Phe, Trp-1999-Cys, Trp-2027-Cys, Trp-2027-Gly, Trp-2027-Leu, Trp-2027-Ser, Ile-2041-Asn, Cys-2088-Arg, and Gly-2096-Ala, in *ACCase* have been identified as the primary cause of *L. chinensis* resistance to Cyhalofop-butyl (Kurniadie et al., 2022; Zhang et al., 2022; Zhao et al., 2022; Liao et al., 2024; Deng et al., 2023; Jiang et al., 2024). In this study, resistant *L. chinensis* populations in Jiangsu Province showed mutations I1781L, W1999C, W2027C/L/S, I2041N, and D2078G in *ACCase*, with no mutations found at the C2088 or G2096 sites. In particular, the W1999C and W2027C mutations were relatively prevalent, whereas I1781L, W2027L/S, I2041N, and D2078G each occurred in a different population. Gene mutations of the target enzyme ACCase are a crucial mechanism underlying the resistance of *L. chinensis* populations to cyhalofop-butyl and fenoxaprop-P-ethyl in the paddy fields of Jiangsu province. Nonetheless, the LELSH-001 and LEHX-002 populations, which showed no *ACCase* amino acid mutations, were categorized as MR to cyhalofop-butyl, suggesting that their resistance may result from non-target mechanisms or enhanced *ACCase* expression. It had been found that the *GSTF1*, *GSTU1*, *GSTU6*, *CYP707A5*, *CYP71C4*, and *CYP 71C1* genes expressed highly in the R population may be responsible for cyhalofop-butyl resistance in *L. chinensis.* (Zhang et al., 2022; Cao et al., 2023). The causes of resistance to cyhalofop-butyl in the LELSH-001 and LEHX-002 populations need to further investigation. All 41 cyhalofop-butyl-resistant *L. chinensis* populations collected in Jiangsu Province were susceptible to oxaziclomefone, with the recommended doses achieving over 90% control efficacy. Similarly, the regular recommended doses of pyraclonil, benzobicyclon, and anilofos showed 100% efficacy in inhibiting biomass accumulation in the 46 geographic populations of *L. chinensis* in Jiangsu Province studied herein, effectively controlling the weed.

Scientific rotation of herbicide types with different mechanisms of action is an effective strategy for controlling noxious weeds and preventing herbicide-resistance development (Evans et al., 2016; Tranel and Wright, 2002). This study revealed that *L. chinensis* populations across different regions of Jiangsu province have developed significant resistance to two members of the aryloxyphenoxypropionate family of ACCase inhibiting herbicides, cyhalofop-butyl and fenoxaprop-P-ethyl. However, all populations were susceptible to the PPO-inhibitor herbicide pyraclonil, the novel bicyclooctane herbicide benzobicyclon, the organophosphorus herbicide anilofos, and the organoheterocyclic herbicide oxaziclomefone. These findings are consistent with those reported by Gu et al. (2021) and Tian et al. (2022), who noted that pyraclonil and benzobicyclon could be used for resistance management in *L. chinensis*. Therefore, using either cyhalofop-butyl or fenoxaprop-P-ethyl alone should be minimized in agricultural practice. Instead, adopting tank mixes and rotation strategies using pyraclonil, benzobicyclon, anilofos, and oxaziclomefone could help delay the development and spread of herbicide resistance in *L. chinensis* populations.

## Data Availability

The raw data supporting the conclusions of this article will be made available by the authors, without undue reservation.
